# Effectiveness of Hypertonic Saline Nasal Irrigation for Alleviating Allergic Rhinitis in Children: A Systematic Review and Meta-Analysis

**DOI:** 10.3390/jcm8010064

**Published:** 2019-01-09

**Authors:** Chia-Ling Li, Hsiao-Chuan Lin, Chien-Yu Lin, Teh-Fu Hsu

**Affiliations:** 1 Department of Nursing, Hsinchu MacKay Memorial Hospital, Hsinchu 30071, Taiwan; m414@mmh.org.tw (C.-L.L.); 6996@mmh.org.tw (H.-C.L.); 2 School of Nursing, National Yang-Ming University, Taipei 11221, Taiwan; 3 Department of Pediatrics, Hsinchu MacKay Memorial Hospital, Hsinchu 30071, Taiwan; mmhped.lin@gmail.com; 4 Department of Emergency Medicine, Taipei Veterans General Hospital, Taipei 11217, Taiwan; 5 Institute of Clinical Nursing, School of Nursing, National Yang-Ming University, Taipei 11221, Taiwan

**Keywords:** hypertonic saline nasal irrigation, nasal irrigation, allergic rhinitis children, nasal symptom

## Abstract

We aimed to explore the efficacy of hypertonic saline nasal irritation (HSNI) for improving nasal symptoms and quality of life, and for decreasing oral antihistamine consumption in children with allergic rhinitis (AR). We conducted a systematic search of PubMed, Medline, Cumulative Index to Nursing and Allied Health Literature, EMBASE, Chinese Electronic Periodicals Service, and Cochrane Library of Controlled Trials databases for prospective randomized, controlled trials assessing HSNI effects in children with AR and published before December 2017. Two authors independently assessed each trial’s quality and extracted data for a meta-analysis. We included four trails comprising 351 patients. HSNI improved patients’ nasal symptom scores (mean difference 1.82 points after treatment; 95% confidence interval (CI), 0.35–3.30; *I*^2^ = 64%; *p* = 0.02) and a significantly lower rescue antihistamine use rate (risk ratio (RR), 0.68; 95% CI, 0.48–0.95; *I*^2^ = 28%; *p* = 0.02). Analyses comparing HSNI with isotonic saline nasal irrigation (ISNI) showed better nasal symptom scores (mean difference, 1.22 points; 95% CI, 1.01–1.44; *I*^2^ = 0%; *p* < 0.001) in patients in the HSNI group, although the antihistamine use (RR, 0.84; 95% CI, 0.64–1.10; *I*^2^ = 0%; *p* = 0.2) and adverse effect rates were similar between groups. Compared with ISNI, HSNI may be a reasonable adjunctive treatment for children with AR.

## 1. Introduction

Allergic rhinitis (AR) is one of the most common diseases in children [1]. It affects 10–30% of children and adults in the United States and in other industrialized countries [2,3]. Nasal congestion, a runny, itchy nose, and sneezing are the cardinal symptoms of AR, and disturbances of sleep and daily functions are common in patients with AR [4,5,6]. The prevalence of AR is similar in Taiwan, with a large disease burden [7]. Co-existence with other allergic diseases, such as atopic dermatitis and asthma, is common. Common AR complications include impaired quality of sleep, daytime fatigue, otitis media, and sinusitis [6,8]. AR remains an important health threat worldwide.

Treatment of AR depends on the age of patients, frequency, and severity of symptoms, and presence of comorbid diseases [9,10,11]. Avoidance of allergens is the cornerstone of successful treatment. Intranasal corticosteroid is an effective treatment with little adverse effects. Combinations of oral or nasal antihistamines, decongestants, cromolyn, or leukotriene-receptor antagonists are the mainstay treatments of severe and refractory AR [4,5,6]. Immunotherapy, subcutaneous or sublingual, may be considered if the usual treatments are unresponsive and the symptoms are not adequately controlled [12]. Allergen-specific tolerance induced by immunotherapy could change the natural course of AR and effectively improve the symptoms. Although these therapies are proven to be effective and safe, non-pharmacological therapy, such as saline irrigation of the nostrils, has been used to alleviate nasal symptoms of AR and may help reduce patient-reported disease severity in both adults and children [13,14]. Saline irrigation is regarded as an adjunctive therapy of AR without severe adverse effects. The entire mechanism of the effect of saline irrigations has not been fully understood, but the clearance of mucus and the removal of airborne allergens and inflammation mediators are believed to contribute to its therapeutic effects [15]. The restoration of the muco-ciliary transport function is also evident in patients using saline irrigation. Saline is an easily available over-the-counter medication, with very few adverse effects reported in patients who use it for nasal irrigation (e.g., nasal bleeding). Therefore, saline irrigation is convenient and effective, and may be used as an alternative treatment for AR [14].

Saline irrigation can be performed by various methods and the optimal volume, pressure, concentration, and delivery devices have not been studied. Isotonic saline (concentration 0.9% sodium chloride) and hypertonic saline (concentration greater than 0.9% sodium chloride) are common solutions used for saline irrigation. Theoretically, hypertonic saline irrigation has a higher osmotic pressure and should exhibit higher efficacy in reducing nasal mucosal edema and in removing airborne allergens and inflammation mediators [16]. However, the optimal concentration of saline solution for irrigation remains unclear. Therefore, we conducted this systematic review and meta-analysis to investigate the efficacy of hypertonic saline nasal irrigation (HSNI).

## 2. Experimental Section

### 2.1. Research Method, Literature Search Strategy, and Results

**Figure 1:**
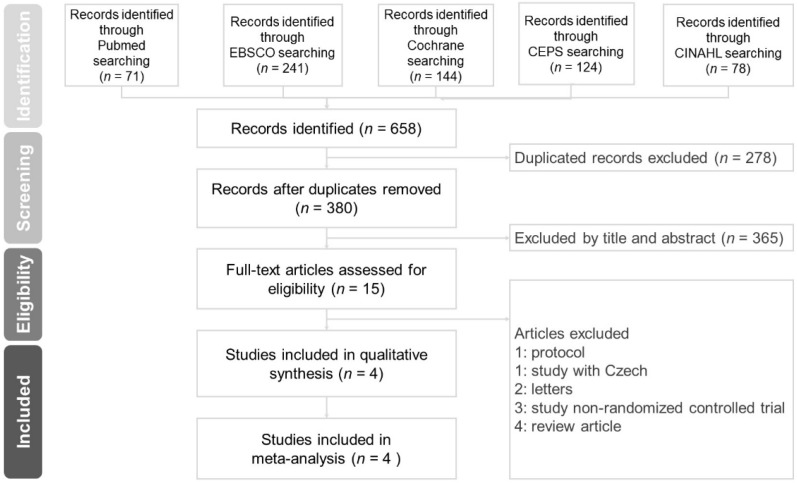
Schematic illustration of the literature search and the study-selection criteria of our study.

We conducted a systematic review and meta-analysis of the medical literature following the Preferred Reporting Items for Systematic Reviews and Meta-Analyses (PRISMA) guidelines to explore the effects of HSNI intervention measures. We identified randomized controlled trials (RCT) investigating the treatment effects of HSNI in patients with AR by searching the Chinese Electronic Periodicals Service, PubMed, Cochrane Central Register of Controlled Trials, MEDLINE, and Cumulative Index to Nursing and Allied Health Literature databases for prospective randomized, controlled trials published before December 2017. We placed no restrictions on publication dates (for either abstracts or full texts). We contacted the corresponding authors of publications with incomplete data or an unavailable full text. If the data were still insufficient, we included only the outcome of the study in the descriptive analysis. We used the following search terms: AR in children, hypertonic saline irrigation, isotonic saline irrigation, rhinorrhea, nasal obstruction, and sneezing. The full search strategy is provided in Table S1. We followed the guidelines outlined in the PRISMA statement for our systematic review. Figure 1 shows the article selection flowchart.

Criteria for the inclusion of studies included the following conditions: (1) the study was an RCT; (2) the study population consisted of children under 18 years of age, and (3) the patients had been diagnosed as having AR. We excluded (1) studies with patients with sinusitis or abnormal nasal structure, and (2) studies on intranasal steroids used during the treatment.

Initially, two independent and professional reviewers (C.-L.L. and H.-C.L.) selected the article titles and abstracts to include. Disagreements were resolved by a third reviewer (C.-Y.L.) whenever necessary. Afterward, we evaluated the full texts of all studies identified as potentially eligible to assess if they fulfilled the inclusion criteria.

### 2.2. Data Extraction and Validity Assessment

Two professionals assessed each study’s quality (risk of bias) using Cochrane’s Risk of Bias Tool (Cochrane Community, London, UK). The review items included: (1) generation of the allocation sequence; (2) concealment of the allocation sequence; (3) blinding of participants and researchers; (4) blinding of outcome assessment; (5) incomplete outcome data; (6) selective reporting; and (7) other biases. The risk of bias tool assessment method includes ‘high risk’, ‘low risk’ and ‘unclear risk’ categories. If review opinions were inconsistent, a third expert was called to review and discuss the quality of the papers and reach an agreement. Figure S1 presents the results of the assessment.

### 2.3. Data Analyses

We performed data synthesis, meta-analysis, and statistical analyses using the Review Manager v5.3.5 software (Cochrane Community, London, UK). We calculated the dichotomous outcomes, risk ratios (RR) and 95% confidence intervals (CIs) based on the data provided by each study. For continuous outcomes, we calculated the mean difference (MD) with 95% CIs. The heterogeneity (*I*^2^) is represented by 0–100%. If *I*^2^ was lower than 50%, it was considered homogeneous, and we adopted a fixed effect model; if *I*^2^ was higher than 50%, we considered it heterogeneous, and adopted a random effect model. A *p*-value < 0.05 was regarded as statistically significant. Lastly, we used a forest plot to indicate the size effect. The primary outcome was nasal symptoms. The secondary outcome measures included rescue antihistamine use, adverse events, and quality of life.

## 3. Results

We identified 658 publications in the literature and assessed 15 studies for eligibility after screening based on the title and abstract contents (Figure 1). We excluded one article written in Czech and 10 articles that were non-randomized controlled trials. We ended up including four studies in our systematic review and meta-analysis (Table 1) [17,18,19,20]. In total, we analyzed data for 351 patients with a male:female ratio of 62.4%:37.6%. The age of participants ranged from 6 to 15 years. Three studies were conducted in Italy and one in Thailand. Two studies compared the efficacy of HSNI with ISNI, one study compared HSNI to no saline irrigation, and one compared HSNI, ISNI, and no saline irrigation. The concentration of HSNI used ranged from 1.25% to 3%, with a frequency of irrigation of two (3 of 4 papers) or three times (1 of 4 papers) per day. The study periods ranged from 3 to 6 weeks. Nasal symptom scores were measured in all studies, and all enrolled studies reported benefits of HSNI. All studies also reported the reduction of antihistamine use in patients using HSNI. Adverse effects such as nasal irritation, burning sensation, or nose bleeding were reported in three studies.

**Figure 2:**
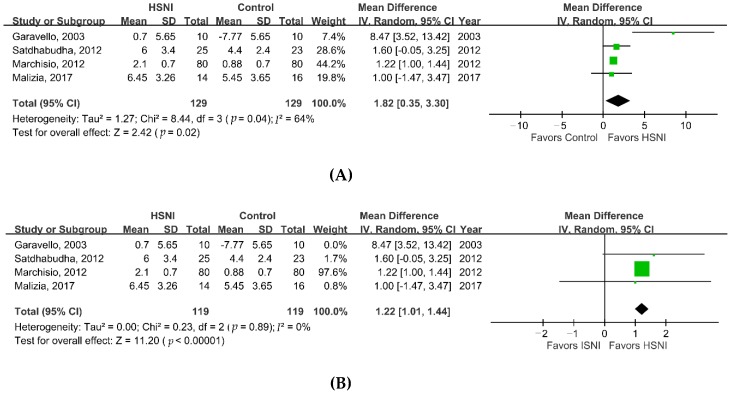
Forrest plot of the improvement of nasal symptom scores in the HSNI and control groups. (**A**) Overall meta-analysis; (**B**) subgroup analysis comparing HSNI and ISNI groups. HSNI, hypertonic saline nasal irrigation; ISNI, isotonic saline nasal irrigation; SD, standard deviation; IV, independent variable; CI, confidence interval.

**Figure 3:**
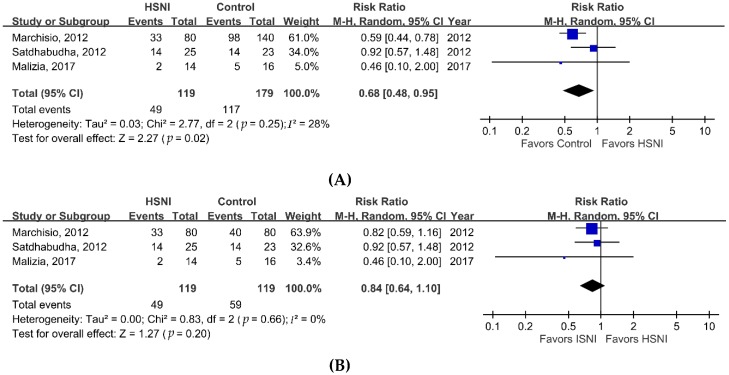
Forrest plot of the rates of rescue antihistamine use in the HSNI and control groups. (**A**) Overall meta-analysis; (**B**) subgroup analysis comparing HSNI and ISNI groups.

**Figure 4:**
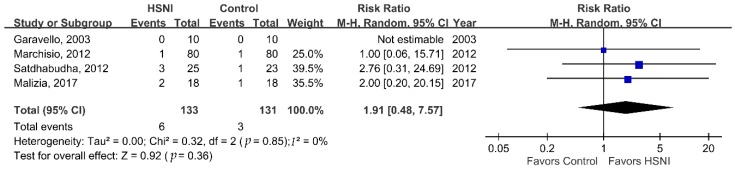
Forrest plot of the adverse effects in the HSNI and ISNI groups.

We performed a meta-analysis to investigate the effectiveness of HSNI for treating AR in children. Compared with the control group, the HSNI group had statistically significant better nasal symptom scores (Figure 2A, MD, 1.82 points; 95% CI, 0.35–3.30; *I*^2^ = 64%; *p* = 0.02). Subgroup analyses comparing HSNI and ISNI treatments showed similar results (Figure 2B, MD, 1.22 points; 95% CI, 1.01–1.44; *I*^2^ = 0%; *p* < 0.001). The comparison of the rate of rescue antihistamine use showed the HSNI group had a significantly lower rate (Figure 3A, RR, 0.68; 95% CI, 0.48–0.95, *I*^2^ = 28%; *p* = 0.02) than the control group. However, subgroup analyses comparing antihistamine use between HSNI and ISNI groups yielded no significant differences (Figure 3B; RR, 0.84; 95% CI, 0.64–1.10; *I*^2^ = 0%; *p* = 0.2). Finally, we found a higher rate of adverse effects in patients using HSNI (Figure 4, 6/133 vs. 3/131 or 4.5% vs. 2.3%), but the differences were not statistically significant (RR = 1.91; 95% CI, 0.48–7.57, *I*^2^ = 0%; *p* = 0.36).

## 4. Discussion

AR is an important disease with a large burden worldwide, and it requires a multistage and multimodality treatment [1,5,6]. The role of HSNI in treating AR was unclear before our systematic review and meta-analysis, but we found evidence supporting its effectiveness. We found four studies investigating the effectiveness of HSNI for treating children with AR, and we identified benefits of HSNI in all of them. Compared with ISNI and no saline irrigation, patients using HSNI had improved nasal symptom scores (MD, 1.82 points; *p* = 0.02). In addition, our subgroup analyses comparing HSNI and ISNI showed significant improvement of nasal symptoms scores in patients using HSNI (MD, 1.22 points; *p* < 0.001). We found a significant reduction of approximately one-third in antihistamine use in patients in the HSNI group (RR, 0.68; *p* = 0.02), but no significant differences between HSNI and ISNI groups (RR, 0.84; *p* = 0.2). Adverse effects in children using HSNI have been of concern, but we found no significant differences between the adverse effects caused by HSNI or by ISNI (RR, 1.91; *p* = 0.36). Therefore, our results support the use of HSNI treatment in children with AR as a more effective adjuvant therapy without more obvious adverse effects than ISNI.

The pathophysiology of AR is complicated, and immune-mediated inflammatory responses play crucial roles [5,21,22,23]. Elevated cytokines have been found in the serum and nasal fluids of patients with AR. Higher IL-5, eotaxin, MIP-1α, and IL-17 levels have been found in the nasal fluid of patients with seasonal AR [24]. Irrigation with saline may remove the inflammatory mediators and decrease the subsequent inflammatory cascades of AR [25,26,27]. Moreover, irrigation may remove sticky secretions and restore the muco-ciliary function [28,29,30,31,32]. These physiological effects may contribute to the observed benefits of saline irrigation [16,33]. Compared with ISNI, HSNI has been proposed to be more powerful in removing mucus and inflammatory mediators [16,33,34,35,36,37,38,39]. Our study provides evidence showing the benefits of HSNI treatment. Drowsiness is common in patients using antihistamines, and the reduction of antihistamine use is a valuable benefit of HSNI use. We found a significant reduction in antihistamine use in patients using HSNI (RR, 0.68; *p* = 0.02) as compared to those not using it, although the benefits were not different between the HSNI and ISNI treatment groups. Adverse effects are a major concern when treating children; however, the adverse effects (nasal irritation, burning sensation, or nose bleeding) reported in our systematic review did not vary between patients treated with ISNI and those treated with HSNI. We analyzed data from a total of 351 patients in our systematic review and nine patients had different kinds of adverse effects (six in the HSNI group and three in the ISNI group), but the differences were not significant. One patient had to withdraw from the study due to adverse effects. Thus, in terms of the alleviation of nasal symptoms and adverse effects, HSNI was superior to ISNI in treating children with AR.

Multimodality treatments are required in treating AR, and saline irrigation is regarded as second-line adjuvant therapy [4,5,6,9,10,11]. Environmental control and avoidance of allergens are important to decrease the frequency and severity of AR, however, complete avoidance is impossible. Pharmacotherapy is frequently needed. Currently, intranasal corticosteroid is the recommended first-line treatment, with good efficacy and safety profile in most guidelines [9,10,11,12,40,41]. For patients with poorly controlled symptoms, combination treatment with oral or nasal antihistamines, decongestants, cromolyn, or leukotriene-receptor antagonists is recommended [9,40,41]. Although these drugs are proven to be effective and safe, long-term drug consumption is usually required due to the fluctuating natural course of AR. Antihistamines may cause drowsiness, irritability, and dizziness; long-term use is discouraged [9,40]. Intranasal corticosteroids relieve symptoms and signs of AR, but concerns regarding children’s growth impairment and the possible long-term adverse effects of increased intra-ocular pressure exist [4,5,6]. Therefore, for patients with inadequately controlled symptoms, additional non-pharmacological therapy, such as saline irrigation, is suggested. While the optimal concentration of saline used for irrigation is unknown, our study demonstrated the superior efficacy of HSNI. The observed adverse effects were similar in HSNI and ISNI groups. Therefore, it’s reasonable to add HSNI treatment in patients with poor responses to intranasal corticosteroid and/or antihistamines, or intolerance of these treatments. Furthermore, in patients with moderate and severe symptoms despite abovementioned therapy, immunotherapy should be considered [9,10,11,12]. Both subcutaneous and sublingual immunotherapy are effective with few adverse effects, and they provide sustained efficacy for more than 10 years after cessation of treatment. Sublingual immunotherapy has lower risk of anaphylaxis, higher compliance and is preferred in pediatric patients. However, the clinical application is not widespread worldwide because of its high cost.

The effects of saline irrigation have been explored in other studies. In Head’s review on nasal saline irrigation in both children and adults, the patient-reported severity of nasal symptoms was reduced without obvious adverse effects [10]. Dichapong et al. compared the effectiveness of HSNI and ISNI for treating patients with sino-nasal diseases, and they found no significant differences between the two groups [30]. We focused on the effectiveness of HSNI for treating patients with AR, and we excluded patients with sinusitis and those using intranasal steroids. The enrolled patients were different and the conclusions also. Although the impact of intranasal steroid use on children’s growth has been shown to be minimal, many parents manifest concerns on the use of intranasal steroids [5,6]. We excluded studies with intranasal corticosteroid use, and we found benefits of HSNI for improving nasal symptom scores and reducing antihistamine use. We discovered no severe adverse effects. In children with AR avoiding intranasal steroids or suffering from complications, HSNI treatment may be a reasonable alternative choice without significant adverse effects. Further studies with large study population are warranted to consolidate the findings and elucidate the benefits and harms of clinical application.

Our study had some limitations. First, although we observed benefits of HSNI in all studies, the protocols of saline irrigation differed. The irrigation frequencies, the HSNI concentrations, and the saline amounts were different. Therefore, further studies are required to determine the optimal regimen of HSNI treatment. Second, the nasal symptom scores used also differed and direct comparisons were unavailable. Although we found nasal symptom score improvements in all studies, the magnitude of improvement and the details need to be clarified. Finally, few studies investigating HSNI are available and they only focused on older children, so the benefits of HSNI treatment in younger children and adults remain to be determined. More study participants are also required to confirm the findings of our study.

## 5. Conclusions

AR is a common disease with troublesome nasal symptoms requiring a multimodality treatment. Although intranasal corticosteroid is an effective and safe therapy, additional treatment is frequently required. Based on our systematic review and meta-analysis, HSNI is a reasonable adjunctive treatment for children with AR, because HSNI provides significantly better improvement in nasal symptoms scores and reduction of antihistamine use than ISNI. The adverse effects in patients using either HSNI or ISNI were mild and similar. Further studies are warranted to elucidate the underpinning mechanisms and confirm the cost-effectiveness of HSNI treatment.

## Figures and Tables

**Table 1:** Characteristics of enrolled trials investigating HSNI treatment on allergic rhinitis.

Author, Year (Ref.)	Country	Participants (M:F)	Age (y/o)	Intervention Design	Concentration	Volume	Daily Frequency	Duration	Outcome	Results	Adverse Effects
Garavello, 2003 [17]	Italy	20 (8:12)	6–12	HSNI:no saline irrigation (1:1)	3%	2.5 mL each nostril	3 times per day	6 weeks	Nasal symptoms score Antihistamine use	Improved nasal symptoms in HSNI group Less antihistamine use in HSNI group	None reported
Marchisio, 2012 [19]	Italy	220 (137:83)	5–9	HSNI:ISNI:no saline irrigation (80:80:60)	2.7%	20 mL	Twice daily	4 weeks	Nasal symptoms score Rhinoscopy Middle ear effusion Reduction of antihistamine use	Both HSNI and ISNI lowered the nasal symptom score HSNI group had a significant reduction of moderate to severe turbinates swelling, adenoidal hypertrophy and bilateral OME HSNI group: fewer antihistamine use	Two children (one in each treatment group)
Satdhabudha, 2012 [20]	Thailand	81 (49:32)	6–15	HSNI:ISNI (40:41)	1.25%	240 mL/time	Twice daily	4 weeks	Nasal symptom score Questionnaire for allergic rhinoconjunctivitis (Rcq-36) Saccharine clearance time	Improvement noted in both groups, but HSNI > ISNI	12% (HSNI) and 5% (ISNI) in 3rd visit
Malizia, 2017 [18]	Italy	30 (25:11)	6–13	HSNI:ISNI (1:1)	3%	5 mL	Twice daily	3 weeks	Nasal symptom score (T5SS) Life quality questionnaires: NCC, PRQLQ, and PSQI Antihistamine use	HSNI group had greater improvement of T5SS NCC decreased in HSNI group; PRQLQ improved in HSNI group; PSQI improved in both groups Reduced antihistamine use was observed in HSNI group	1 child in ISNI (6%) versus 2 children in HSNI (12%)

Abbreviations: HSNI, hypertonic saline nasal irrigation; ISNI, isotonic saline nasal irrigation; NCC, nasal cytology counts; OME, otitis media with effusion; PRQLQ, paediatric rhinoconjunctivitis quality of life questionnaire; PSQI, Pittsburgh sleep quality index; Rcq-36, questionnaire for Thai allergic rhinoconjunctivitis patients; T5SS, total 5 symptom score; Ref, reference; M, male; F, female; y/o, years old.

## Supplementary Materials

The following are available online at http://www.mdpi.com/2077-0383/8/1/64/s1, Table S1, Search strategy for systematic review and meta-analysis; Figure S1, Risk of bias summary: review-authors’ assessment about risk of bias items in each study.
