# Supplementary material for: Effectiveness of Hypertonic Saline Nasal Irrigation for Alleviating Allergic Rhinitis in Children: A Systematic Review and Meta-Analysis
Source: J Clin Med. 2019 Jan 9;8(1):64. doi: 10.3390/jcm8010064 (PMC6352276; doi:10.3390/jcm8010064)
Supplement: Supplementary file 1 [file jcm-08-00064-s001.zip › supplementary/Supplementary table 1.docx]

Table S1. Detailed search strategy of present study

| **PICO** | **PICO** |
| --- | --- |
|  | **關鍵字、同義字、*MeSH terms*** |
| **P** | *allergic rhinitis*, allergic rhinitides in children |
| **I** | hypertonic saline irrigation, nasal (spray OR lavage) (saline OR sodium chloride OR saline solution), intranasal administration (saline OR sodium chloride OR saline solution), nasal saline irrigation*, nasal lavage* |
| **C** | routine care, no nasal irrigation, isotonic saline nasal (spray OR lavage OR irrigation) |
| **O** | ***Primary outcome***: rhinorrhea, nasal obstruction, sneezing, (nasal OR orophraryngeal OR ocular) itching,  ***Secondary outcome***: rescue antihistamines use , adverse events (*epistaxis,* nasal bleeding, nose bleed, nasal irritation or burning), quality of life |
