# Supplementary figures and images for: Effectiveness of Hypertonic Saline Nasal Irrigation for Alleviating Allergic Rhinitis in Children: A Systematic Review and Meta-Analysis
Source: J Clin Med. 2019 Jan 9;8(1):64. doi: 10.3390/jcm8010064 (PMC6352276; doi:10.3390/jcm8010064)

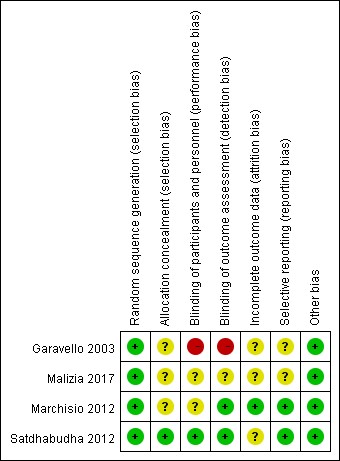

Supplement: Supplementary file 1 [file jcm-08-00064-s001.zip › supplementary/Figure S1.jpg]
